# Oxidative stress signaling in the pathogenesis of diabetic cardiomyopathy and the potential therapeutic role of antioxidant naringenin

**DOI:** 10.1080/13510002.2023.2246720

**Published:** 2023-09-25

**Authors:** Nan Xu, Siqi Liu, Yongqiang Zhang, Yujing Chen, Yumei Zuo, Xiaoqiu Tan, Bin Liao, Pengyun Li, Jian Feng

**Affiliations:** a Department of Cardiology, The First People's Hospital of Neijiang, Neijiang, People’s Republic of China; b Department of Cardiology, The Affiliated Hospital of Southwest Medical University, Key Laboratory of Medical Electrophysiology, Ministry of Education and Medical Electrophysiological Key Laboratory of Sichuan Province, Institute of Cardiovascular Research, Southwest Medical University, Luzhou, People’s Republic of China; c Department of Cardiovascular Surgery, The Affiliated Hospital of Southwest Medical University, Metabolic Vascular Diseases Key Laboratory of Sichuan Province, Luzhou, People’s Republic of China

**Keywords:** Diabetic cardiomyopathy, naringenin, oxidative stress, signal pathways, Nrf2, REAGE, NOX, NF-κB

## Abstract

Diabetes mellitus (DM) is one of the most prevalent metabolic disorders that poses a global threat to human health. It can lead to complications in multiple organs and tissues, owing to its wide-ranging impact on the human body. Diabetic cardiomyopathy (DCM) is a specific cardiac manifestation of DM, which is characterized by heart failure in the absence of coronary heart disease, hypertension and valvular heart disease. Given that oxidative stress is a key factor in the pathogenesis of DCM, intervening to mitigate oxidative stress may serve as a therapeutic strategy for managing DCM. Naringenin is a natural product with anti-oxidative stress properties that can suppress oxidative damage by regulating various oxidative stress signaling pathways. In this review, we address the relationship between oxidative stress and its primary signaling pathways implicated in DCM, and explores the therapeutic potential of naringenin in DCM.

## 1. Introduction

Diabetes Mellitus (DM) is a global public health issue that poses a considerable threat to human health. Factors such as economic development, industrialization, changes in lifestyle, and ageing have contributed to the gradual increase in the incidence of DM. According to the International Diabetes Federation (IDF) Global Diabetes data released in 2017, there are currently 425 million adults with diabetes worldwide, and this number is expected to rise to 631 million by 2045 [1,2]. DM is a chronic metabolic disorder characterized by elevated plasma glucose levels [3]. The autoimmune destruction of islet beta cells, leading to insufficient insulin production, and endogenous resistance of body cells to insulin action are the primary causes of DM-related chronic hyperglycemia [4,5]. The morbidity and mortality of cardiovascular diseases in diabetic patients are closely related to coronary artery disease and heart remodeling, which are the most common cardiac complications of DM [6,7].

Multiple studies have shown that oxidative stress is a major upstream event in the development of diabetic complications and insulin resistance, inducing pathophysiological mechanisms and initiating a series of harmful pathways leading to insulin resistance and diabetes [8–10]. Insulin resistance can lead to a variety of changes, such as increased systemic blood pressure, elevated triglyceride levels, and decreased HDL levels, which are risk factors for vascular dysfunction [11]. In addition, whole-gene expression profiles in epicardial adipose tissue from patients with coronary artery disease suggested alterations in genes involved in oxidative stress [12]. Oxidative stress plays an important role in the development and pathophysiology of diabetes and its various complications through lipid peroxidation, DNA damage and mitochondrial dysfunction [13,14]. It was reported that reducing oxidative stress by oxidase inhibitors could improve glucose metabolism in mice with diabetes [15]. Diabetic Cardiomyopathy (DCM), as an independent cardiac complication of diabetes, is one of the leading causes of death in patients with diabetes. Long-term hyperglycemia can cause oxidative stress, inflammation, myocardial fibrosis, apoptosis and mitochondrial damage in cardiomyocytes, affecting the diastolic function of the heart in the early stage, and the diastolic function in the late stage, and finally developing into congestive heart failure [16]. DCM contributes to nearly 80% of mortality in patients with DM among all complications of diabetes [17,18]. Therefore, new therapeutic strategies that can effectively intervene in the occurrence and development of DCM can bring benefits to diabetic patients.

In the search for better treatment strategies, researchers have turned to natural product extracts with widespread availability and safety. Therefore, in recent years, the research into natural products for DCM has gradually increased, and naringenin, as one of them, has attracted considerable attention. Naringenin, a natural flavonoid mainly found in Rutaceae, has received much attention in the treatment or prevention of various disorders, including diabetes, cardiac diseases, atherosclerosis and metabolic diseases. Modern medical studies have confirmed that naringenin have significant therapeutic potential in several diseases through anti-oxidative, anti-inflammatory, anti-apoptotic, and anti-fibrosis actions, as well as participating in regulating glucose and lipid metabolism [19–24]. In this review, the signaling pathways involved in the development of DCM, and the antioxidant activities of naringenin, as well as the recent advances in the use of naringenin for the prevention and treatment of DCM have been reviewed, with the intention of identifying appropriate targets and new antioxidant therapies for DCM.

## 2. Development of diabetic cardiomyopathy

In 1972, Rubler et al. reported a special type of cardiomyopathy in diabetic patients and defined it as DCM [25]. DCM refers to the clinical condition of abnormal myocardial structure and function in diabetic patients without other cardiovascular diseases, such as coronary heart disease, hypertension, and significant valvular disease. It can be considered as a specific myocardial injury. In the early stages of diabetic cardiomyopathy, metabolic disorders promote structural and functional adaptations of the heart. These include impaired insulin metabolic signaling, environmental insulin excess, impaired glucose uptake, and mitochondrial dysfunction. These common metabolic disorders promote cardiac remodeling, fibrotic diastolic dysfunction, and ultimately lead to reduced ejection fraction in diabetic patients [26,27].

**Figure 1.**
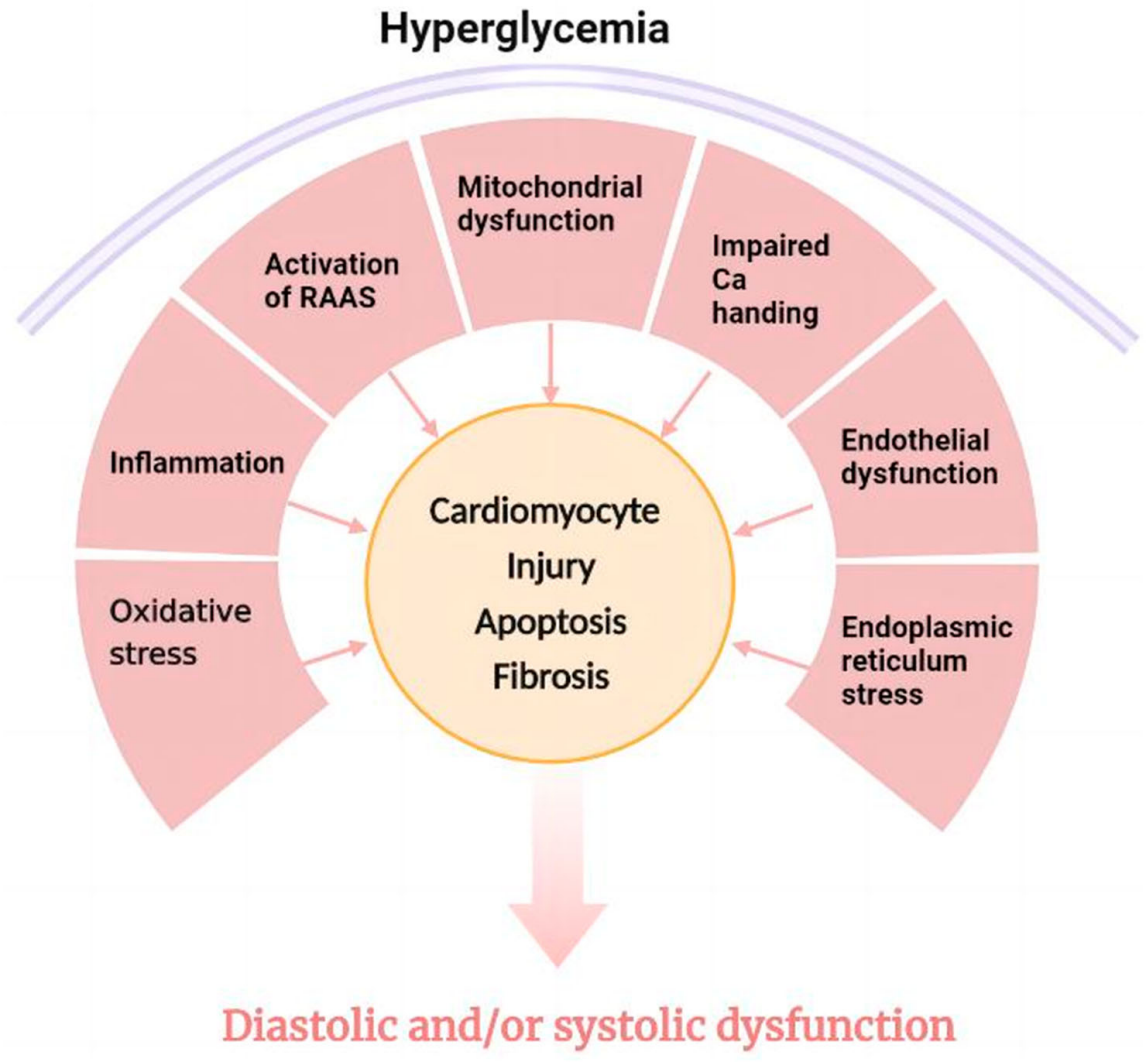
The pathophysiological mechanism of diabetic cardiomyopathy. The occurrence and development of diabetic cardiomyopathy involve multiple mechanisms. These include mitochondrial dysfunction, oxidative stress, inflammation, calcium processing disorders, endoplasmic reticulum stress, microvascular dysfunction, renin-angiotensin-aldosterone system (RAAS) activation, and multiple cardiometabolic abnormalities. Both are involved in structural remodeling and functional defects of diabetic myocardium.

In T1DM and T2DM experimental animal models, the diastolic and contractile functions of cardiomyocytes were decreased, accompanied by the reduction of cardiomyocyte contractile function and the changes of specific cardiomyocyte proteins [28]. A variety of mechanisms are involved in the development of DCM (Figure 1), including mitochondrial dysfunction, oxidative stress, inflammation, impaired calcium processing, endoplasmic reticulum stress, microvascular dysfunction, renin-angiotensin-aldosterone system (RAAS) activation, and multiple cardiometabolic abnormalities [29,30]. Here we mainly focused on oxidative stress-related signaling pathways in the development of DCM.

## 3. Role of oxidative stress in diabetic cardiomyopathy

Oxidative stress results from an imbalance between reactive oxygen species (ROS) production and oxidative scavenging systems [31]. Excessive ROS production can lead to myocardial inflammatory damage, Causes include lactate dehydrogenase (LDH), creatine kinase isoenzyme (CK-MB), cardiac troponin-T (cTn-T) and cardiac troponin-I (cTn-I) increased [32–34]. The intensity of these myocardial injury markers is positively correlated with the degree of myocardial injury and heart failure [35,36]. In animal models of heart failure, the impairment of systolic and diastolic cardiac function was associated with increased ROS levels in cardiomyocytes [37,38]. ROS generation and activation of the antioxidant system were detected in cardiomyocytes of leptin receptor deficient db/db mice, severe hyperglycemia, and type 2 diabetes [39,40]. Excessive ROS production caused damage to cellular proteins, nucleic acids, lipids and other biomolecules [41,42], and also destroyed cellular antioxidant defense system, resulting in disturbance of apoptosis-related molecular signaling pathways [43,44].

ROS can also inactivate JNK-inactivating phosphatases, leading to JNK activation, followed by cytochrome c release, caspase-3 activation and apoptosis [45]. After massive death of normal cardiomyocytes due to myocardial injury, myocardial fibroblasts proliferate and differentiate into myofibroblasts, promoting extracellular matrix remodeling and myocardial fibrosis, eventually leading to myocardial structural changes and heart failure [46]. Hyperglycemia-induced increases in glucose autoxidation, protein glycosylation, and oxidative degradation of glycosylated proteins in a diabetic setting lead to overproduction of ROS [47]. ROS can be triggered by nuclear factor-κ-gene binding (NF-κB), and inflammatory factors such as TNF-α, interleukin-6 (IL-6) and interleukin-10 (IL-10) can promote the inflammatory damage of myocardial cells [48,49]. Therefore, when oxidative stress signaling pathways are activated, ROS production must be strictly regulated and reduced to alleviate oxidative damage or stress [50]. Importantly, several studies have shown the role of antioxidant stress interventions in diabetic heart disease, and clearance of ROS can limit or prevent cardiac dysfunction in animal models of diabetes [51,52].

## 4. Oxidative stress signaling pathways in DCM

Several oxidative stress-related signaling pathways, such as nuclear factor erythroid 2-related factor 2 (Nrf2), advanced glycosylation end products (AGEs), nicotinamide adenine dinucleotide phosphate (NADPH) oxidase (NOX), and nuclear factor kappa-light-chain-enhancer of activated B cells (NF-κB), have been shown to be associated with DCM.

### 4.1 Nrf2 signaling

**Figure 2.**
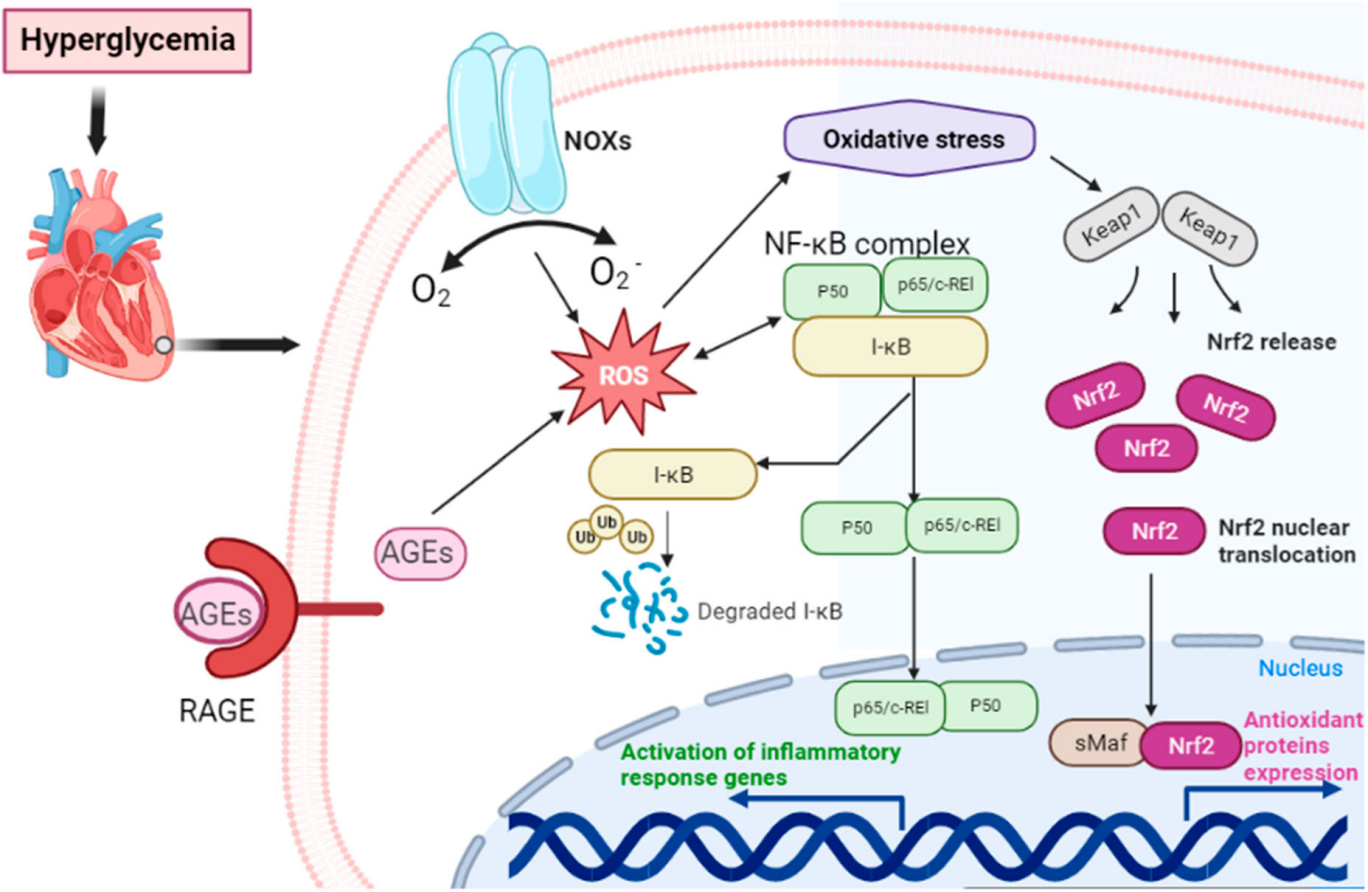
The role of different signaling pathways in DCM. Diabetes induces the formation of AGEs, which can simultaneously induce the activation of NOXs, RAGE and NF-κB to induce the excessive production of ROS, followed by the enhancement of oxidative stress. The activation of NOXs, RAGE and NF-κB can also cause and inflammation and oxidative damage. Oxidative stress can cause cardiomyocyte injury, apoptosis, extracellular matrix accumulation, myocardial fibrosis, remodeling, and dysfunction, all of which are characteristic of DCM. Nrf2 signaling pathway to produce antioxidant factors such as SOD and NQO1 to reduce oxidative damage. AGEs: advanced glycosylation end products; RAGE: receptor for AGEs; NF-κB: nuclear factor kappa-light-chain-enhancer of activated B cells; NOX: NADPH oxidase; Nrf2: nuclear factor erythroid 2-related factor 2; ROS: reactive oxygen species; DCM: diabetic cardiomyopathy.

Nrf2 belongs to the Cap’n’Collar (CNC) subfamily of basic leucine zipper (bZip) transcription factors, which comprises nuclear factor erythroid-derived 2 (NFE2) and Nrf1, Nrf2, and Nrf3. Nrf2 possesses seven conserved Nrf2-ECH homology (Neh) domains with different functions. Under physiological conditions, Nrf2 combines with Kelch-like ECH-associated protein 1 (KEAP1) to inhibit its activity [53]. Under the appropriate stimulation by physical and chemical factors, Nrf2 uncouples to Keap1 and translocates in the nucleus, where Nrf2 binds to antioxidant response elements (ARE) and initiates the transcription and expression of ARE-regulated antioxidant enzyme genes [54]. Activated Nrf2 regulates the transcription of antioxidant enzymes and induces a series of antioxidant transcriptases, including NADPH quinone oxidoreductase (NQO1), glutathione-S-transferase (GST), heme oxygenase-1 (HO-1), Glutathione Peroxidase (GPx), Glutathione Reductase (GR), γ-glutamylcysteine synthethase(γ-GCS), superoxide dismutase (SOD), catalase (CAT). Thus, it can quickly and effectively remove the excess ROS produced in the body to reduce oxidative stress [55,56] (Figure 2).

Several studies have also demonstrated that Nrf2 and its downstream signaling pathways play a key role in the prevention of high glucose-induced DCM and other oxidative damage caused by cardiovascular diseases. For example, it was demonstrated that Nrf2 and its downstream antioxidant protein expression were inhibited in H9C2 myocardial cells treated with high glucose. This study showed inhibition of the antioxidant stress mechanism in high glucose environment. Meanwhile, curcumin intervention activated the Nrf2/HO-1 signaling pathway in a DM rat model, which reduced the oxidative damage to cardiomyocytes [57]. This study suggests that the Nrf2 signaling pathway plays a role in DCM progression, but the experimental design did not include a comparison of the control group with Nrf2 gene knockout group. Additionally, inhibition of soluble epoxide hydrolase AUDA (compound 43) could stimulate the Nrf2 signaling pathway to reduce the progress of DCM. However, AUDA did not show these effects in the Nrf2 knockout DCM model. The results indicated that transcriptional activation of Nrf2 could affect the development of DCM [58]. These studies proved that Nrf2 signaling pathway played a role in the progression of DCM, and the up-regulated expression of Nrf2 is beneficial against the development of DCM.. Additionally, another study not only measured the expression of oxidative stress-related substances, but also proved that Nrf2 signaling pathway inhibited the progression of DCM by alleviating oxidative stress [59]. Moreover, Nrf2 knockout mice were more likely to develop severe cardiomyopathy in streptozotocin (STZ)-induced diabetes than wild-type mice in animal experimental models [60]. Natural or synthetic Nrf2 activators have therapeutic effects in DCM model animals. All these studies confirmed the protective role of Nrf2 in DCM [61]. Therefore, Activation Nrf2 signaling pathway is considered a promising therapeutic strategy for the treatment of DCM.

### 4.2 RAGE signaling

RAGE (receptor of advanced glycation end products) is a multiligand surface receptor found in a large number of cell types, including endothelial cells, nerve cells, cardiomyocytes, mesangial cells, and cells of the immune system [62]. The binding of AGEs with their receptors (RAGE) induces a variety of microvascular and macrovascular complications of diabetes by forming of cross-links between molecules in the basement membrane of the extracellular matrix, leading to cardiovascular damage. In patients with diabetes, a large number of AGEs are formed and deposited in the myocardial microvascular endothelium and extracellular matrix by non-enzymatic glycation reaction under long-term continuous high glucose environment [63]. AGEs can change the structure of proteins, cross-link collagen molecules, and directly cause irreversible damage to myocardial microvascular endothelial cells [64,65]. In addition, AGEs bind to RAGE and activate AGE-RAGE signaling pathways, causing oxidative stress, inflammatory response, and protein kinase C activation, which further promote ROS production [66,67].

It has been demonstrated that AGE and ROS jointly promote myocardial cell damage and fibrosis, which is the pathological basis of heart failure [68] (Figure 2). It was reported that the calcium transient amplitude and calcium content of sarcoplasmic reticulum greatly decreased in AGE-treated rat cardiomyocytes, while the ROS generation in cardiomyocytes increased significantly, suggesting that RAGE signaling pathway is essential for the pathophysiological development of DCM [69]. Similarly, in a study involving 2426 participants, it has been demonstrated that AGEs accumulation is an independent risk factor for heart failure, and the study also indicated that increased myocardial AGEs accumulation affected diastolic function in patients with diabetes [70]. However, there have been inconsistent results in basic and clinical trials related to AGEs and heart failure. It was reported that AGE did not independently predict heart failure [71]. AGE inhibition failed to improve exercise tolerance in heart failure patients with systolic dysfunction [72]. The discrepancy may be attributed to the different classification of heart failure by left ventricular ejection fraction (LVEF) in the selection of research participants More clinical studies are needed to reveal the effects of AEG on heart failure patients with diabetes, and more trials are necessary to explore the related mechanisms.

### 4.3 NOX signaling

NOX is an enzyme that produces superoxide or hydrogen peroxide from molecular oxygen. It was first discovered on the membrane of phagocytes, and its primary function is to produce ROS to eliminate pathogens in immune defense. NOX is composed of two membrane subunits, gp91phoxand p22phox, forming the flavocytochrome b558, catalytic core of the enzyme, the cytosolic subunits p67phox, p47phox and p40phox, and the small GTPase Rac. Over the years, seven isoforms of NOXs (NOX1 – NOX5, dual oxidase 1 (DUOX1) and DUOX2) have been identified. Among them, NOX1, NOX2 and NOX4 were highly expressed in diabetic hearts [73,74]. High glucose promoted the recruitment of NOX catalytic subunits and NOX activation. This activation led to the generation of superoxide, which in turn promoted mitochondrial ROS production in a positive feedback loop [75,76]. In the DCM rat treated with a traditional Chinese medicine ShengMai-San [77], the researchers suggested that the expression levels of NOX2 and NOX4 in the myocardium of diabetic rats were significantly increased. Down-regulation of NOX2 and NOX4 expression after intervention reduced cardiomyocyte apoptosis and fibrosis in rats. Therefore, it was demonstrated that NOX signaling pathway was involved in the progression of DCM. Another study also showed that inhibition of NOX signaling pathway could reduce myocardial oxidative damage by regulating the interaction of NOX2, NOX4 and ROS, thereby acting as a potential therapeutic target for the prevention and treatment of DCM [78] (Figure 2). All these studies suggested that inhibition of NOX signaling may be a potential treatment target for DCM.

### 4.4 NF-κB signaling

Excessive ROS activates NF-κB, a redox-sensitive protein complex that plays a central role in inflammation. Activated NF-κB promotes the transcription and release of proinflammatory mediators such as interleukin-6 (IL-6) and tumor necrosis factor-α (TNF-α), which causes myocardial inflammation [79]. Several studies have demonstrated that inhibition of NF-κB signaling pathway can reduce inflammation and oxidative stress, and reduce fibrosis, hypertrophy and apoptosis *in vitro* and *in vivo* [80] (Figure 2), thereby preventing DCM. In high glucose-treated H9c2 cells, researchers have demonstrated that NF-κB signaling plays a central role in high glucose-induced myocardial injury by experimentally inhibiting the NF-κB inflammatory pathway [46]. In streptozotocin-induced DCM rat model and high glucose stimulated H9C2 model of DCM, inhibition of NF-κB P65 nuclear translocation resulted in improved cardiac function [81]. Studies have also shown that NF-κB pathway inactivation is an effective treatment target for DCM [82,83], and NF-κB signaling pathway can also cooperate with AGEs and NOX signaling pathways to cause more severe oxidative damage [84,85]. These studies suggest that the pathological basis of DCM may be caused by the interaction of multiple signaling pathways. However, more detailed studies are needed to explore the relationship between these signaling pathways.

## 5. Role of naringenin, an antioxidant, on diabetic cardiomyopathy

Oxidative stress plays critical role in the development of DCM, and anti-oxidative stress is essential for the treatment of DCM. To date, a number of studies have demonstrated that several natural products could attenuate the pathological progression of DCM via an anti-oxidative stress mechanism [86,87]. However, the exact target through which these compounds exerted the functions remained unclear. Additionally, despite the reports of animal and cell experiments on the effects of these natural products on DCM, the use of these products in clinical trials, to date, has not been conducted.

**Figure 3.**
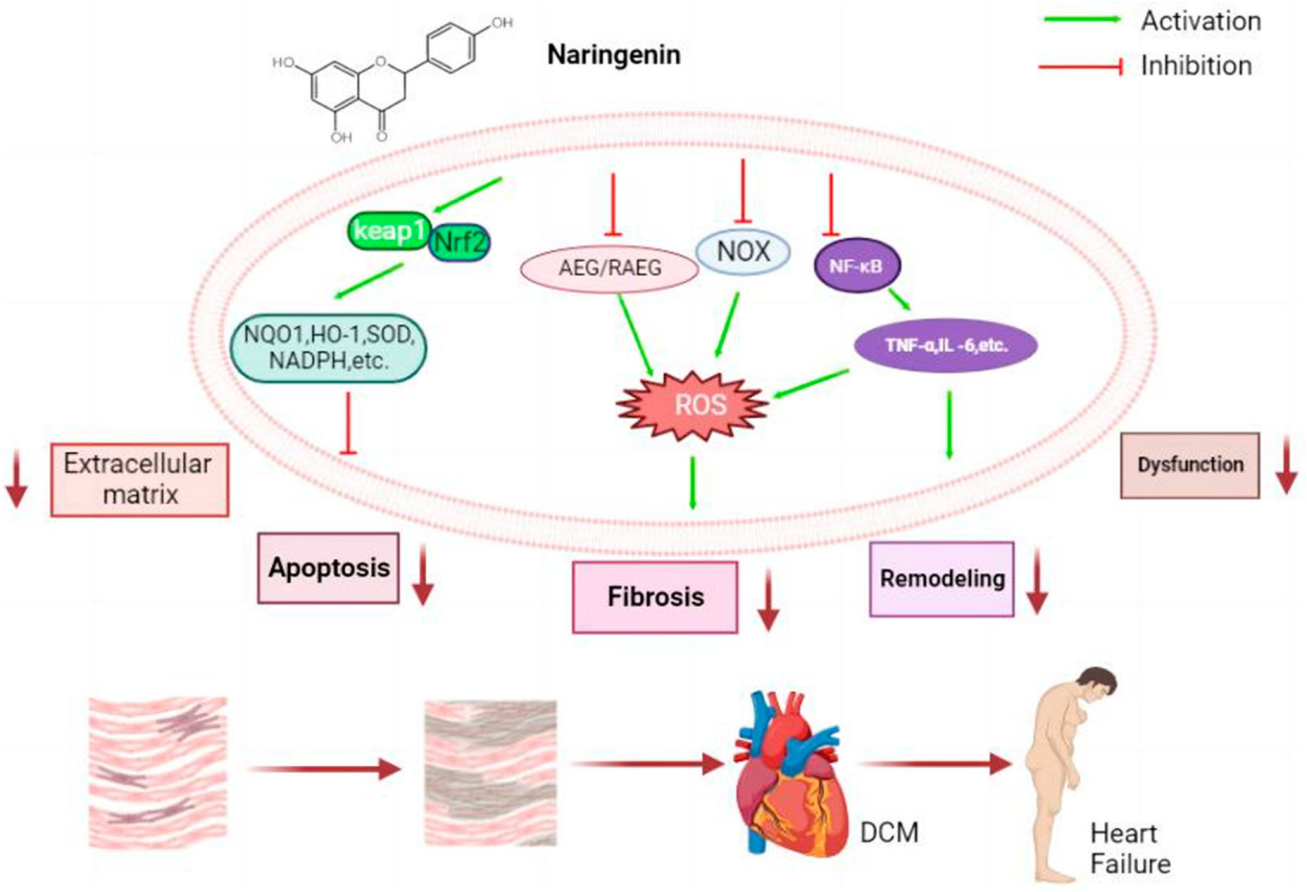
The role of naringenin in DCM. Naringenin can inhibit the activation of ARG/RAGE, NOXs and NF-κB signaling pathways, and promote the activation of Nrf2 signaling. Naringenin, by altering the expression of these signaling pathways, can reduce the structural changes of cardiac muscle cells in many ways and alleviate heart failure.

Naringenin is a polyphenolic phytochemical that has been widely found in a variety of herbs and fruits, including oranges, lemons, grapes, oranges, grapefruit, bergamot, tomatoes, cocoa, as well as cherries. As a natural product extract, naringenin has been mentioned in many antioxidant studies. It has gained increasing attention due to its positive biological activities such as anti-diabetes, anti-inflammation and anti-oxidation [88] (Figure 3). Even though naringenin has not been used in clinical experiments, a number of animal and cell experiments have proved its anti-hyperglycemia and anti-oxidative stress effects in diabetes [89]. For instance, naringenin could protect STZ-induced pancreatic MIN6 cells against oxidative stress by up-regulating the activity of Nrf2/HO-1 signaling pathway *in vivo* and *in vitro* [90]. Additionally, another study showed that naringenin could increase the expression of Nrf2 and its downstream target genes by activating the Nrf2/ARE signaling pathway in neurons, and reduce neuronal oxidative stress injury caused by ischemia-reperfusion [91]. This study is consistent with the findings of Yang Y.et al [92]. Inhibition of Nrf2 signaling pathway reduced the protective effect of naringenin in cardiac fibroblasts and vascular endothelial cells [93,94]. In another study, naringenin was found to activate the Nrf2 signaling pathway and inhibit the NF-κB signaling pathway in DCM rat models, suggesting that naringenin may affect multiple signaling pathways involved in the formation of DCM simultaneously [95].

Similar to Nrf2 signaling pathway, NF-κB signaling has repeatedly been shown to be involved in cellular oxidative stress. It was found that in diabetic rat, naringenin was able to down-regulate the expression of NF-κB signaling pathway and its downstream inflammatory factors, which reduced inflammation and apoptosis of myocardial cells [20]. Naringenin was found to have a dual inhibitory effect on the formation of AGEs and age-induced cellular oxidative stress. The protective effects of naringenin on age-induced oxidative stress and inflammation were detected in RAW267.4 cells treated with naringenin [96]. In a separate study, researchers also demonstrated that naringenin inhibition of AGEs formation reduced glycosylation of human serum albumin [97]. Many other studies have investigated the inhibitory effect of phenolic compounds on the formation of AGEs in chemical or biological models and suggested that their effect may be related to their antioxidant activity or their ability to capture active carbonyl substances [98,99].

Studies of naringenin on NOX signaling pathway mainly focused on the cardiovascular system. In the study of lipopolysaccharide-stimulated mouse macrophages, naringenin can effectively inhibit the proinflammatory response of macrophages and reduce the expression of NOX2 [100]. Furthermore, in studies of cisplatin-induced renal injury, naringenin intervention significantly reduced NOX4 protein and mRNA levels *in vivo*, limiting NOX4-dependent oxidative stress to play a key role in cisplatin-induced renal injury, programmed cell death, and inflammation [101]. Although no studies on the RAGE and NOX signaling pathways have been found in the DCM model, considering the effect of naringenin on the anti-oxidative stress of these two signaling pathways in other organs, it is expected that there will be relevant studies on the intervention of naringenin in the RAGE and NOX signaling pathways in cardiomyocytes [102,103].

## 6. Conclusions and perspectives

Preclinical studies have provided substantial evidence that oxidative stress-triggered signaling pathways play an essential role in the pathogenesis of DCM. The pathological process of DCM can be mitigated by inhibiting the pro-oxidant role of NF-κB, NOX and RAGE signaling pathway, or promoting the anti-oxidant role of Nrf2 signaling pathway in oxidative stress. However, it is unclear whether coordination occurs between these regulation pathways in DCM, the precise role that oxidative stress plays in the development and progression of DCM has not been fully investigated.

Current recommended treatments for DCM patients focused on anti-diabetic, anti-inflammatory, and anti-ventricular remodeling. But in fact, oxidative damage is already present from the beginning of prediabetes. Anti-oxidative stress intervention may begin to benefit from prediabetes. For example, studies have shown that long-term consumption of broccoli sprouts in obese and dysglycemic patients improves fasting blood glucose and glycated hemoglobin (HbA1c) levels, and reduces oxidative products and inflammatory markers in the body[104]. Naringenin has better antioxidant activity than ascorbic acid [105], and can also activate the expression of antioxidant enzymes such as SOD, NQO1 and HO-1 [92,93]. Furthermore, most ongoing research has focused on natural products, suggesting that their therapeutic value merits more attention. As a natural flavanone, naringenin may exert beneficial effects as an anti-oxidative stress and hypoglycemic agent against the complications for the treatment of DCM. Studies on the safety and pharmacokinetics of naringenin have proposed that doses of 150–900 mg of naringenin are safe for healthy adults, and the serum concentration is proportional to the administered dose. The intake of 300 mg naringenin twice daily may cause a physiological response [106]. Clinical studies of naringenin in the treatment of cardiac risk factors such as obesity and arteriosclerosis have also proved that naringenin is safe and beneficial when administered to humans [107–109]. It is reasonable to expect that naringenin will become a new treatment strategy for DCM. However, the mechanism, specificity, bioavailability and drug interactions of naringenin need to be further studied before it can be used in clinical treatment.

## Data Availability

The data that support the findings of this study are openly available in pubmed at https://pubmed.ncbi.nlm.nih.gov/, reference number [1–109].
